# Peno-scrotal limphedema with giant hydrocele - surgical treatment particularities

**Published:** 2009

**Authors:** Dan Mischianu, Ioan Florescu, Victor Madan, Cristian Iatagan, Ovidiu Bratu, Anca Oporan, C Giublea

**Affiliations:** * “Dr. Carol Davila” Central Military Emergency Hospital - Department of Urology; ** “D. Bagdasar” Hospital - Department of Plastic and Reconstructive Surgery

## Abstract

**Introduction**:

The necessity for complex and multidisciplinary approach of “border” surgical pathology has unanimously been agreed upon for such a long period of time, its advantages becoming even more obvious in rare, particular cases.

**Patients and methods**:

We report the case of a 39 year-old man diagnosed with lymphangiomatosis back in his childhood. He is admitted with a giant pseudotumoral scrotal mass presenting an important scrotal enlargement (40/35 cm).

Physical examination, blood tests, ultrasound, IVP, abdominal and chest CT, psychiatric and plastic surgery evaluation established the diagnosis: peno-scrotal lymphedema with gigantic hydrocele and depressive disorder. Taking into account the important enlargement of the scrotum associated with the alteration of the local skin, we decided to form a mixed surgical team: urology - plastic and reconstructive surgery.

We performed bilateral surgical therapy of hydrocele with partial excision and eversion of sac edges, excision of peno-scrotal skin and subcutaneous tissue surplus. At the end we made a reconstruction by using a partial-thickness graft from the normal skin of the left thigh.

**Results**:

Spinal anaesthesia was sufficient in order to perform a qualitative complex surgery. Intra and postoperative course was uneventful with minimal blood loss.

**Conclusion**:

Rare cases like this one clearly reveal the advantages of a multidisciplinary surgical team by combining usual surgical procedures from different specialities that could lead to spectacular results.

## Introduction

Lymphedema is an abnormal collection of protein-rich fluid in the interstitium and, regardless of etiology, is caused by inadequate lymphatic drainage leading to a chronic inflammatory process of hypertrophy and hyperplasia of connective tissue.

Peno-scrotal lymphedema is a condition which leads to the progressive enlargement of the scrotum and penis causing significant psychical discomfort for patients, making both sexual intercourse and urination act difficult or even impossible, impairing movement and proper hygiene of the perineal region.

The lymphedema is classified into primary, idiopathic - when it is caused by lymphatic malformation and could be congenital (Milroy disease), lymphedema praecox (clinical manifested before 35 years old), and lymphedema tarda (after 35 years old).

Secondary lymphedema is due to an acquired obstruction and most frequently is the result of a parasite spreading disease – filariosis. The larvae of *Wuchereria bancrofti* warm (that causes filariosis) transmitted by mosquito bites migrate to the lymphatic tissue of the scrotal region causing lymphedema [**1**].

But the second type of lymphedema can result out of inflammatory or infectious diseases such as lymphogranulomatosis, tuberculosis or bacterial cellulitis.

The most common etiologic malignancy is the prostate cancer with metastases inguinal lymph nodes.

Surgical procedures, especially inguinal lymphadenectomy and radiation therapy in the pelvic region, can also be important causes. Literature mentions only one case of peno-scrotal lymphedema after complicated circumcision [**1**], [**2**], [**4**].

Renal, hepatic or cardiac diseases with secondary hypoproteinemia can also lead to peno-scrotal lymphedema. However, rarely but possibly, the lymphedema may be associated with Crohn disease, sarcoidosis and rheumatoid arthritis.

The peno-scrotal lymphedema secondary to silicon subcutaneous or intradermic penian self injection is also described [**3**].

There are about 30 cases of peno-scrotal lymphedema listed on the internet, on the specialized sites, presenting different types of etiologies. However, the rarity of the cases and the surgical methods make the reports useful.

## Case report

We report the case of a 39-year-old man who was diagnosed with congenital lymphangiomatosis at the age of 12 when he underwent diagnostic intra-abdominal lymph node excision.

**Fig. 1:**
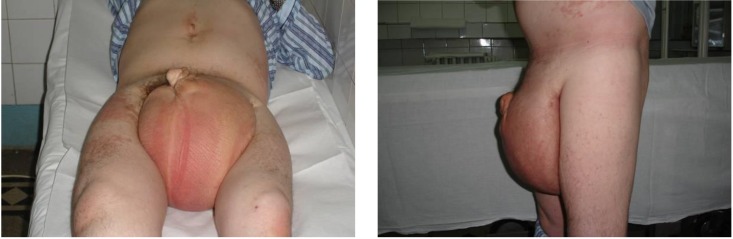
Preoperative appearance – important enlargement of the scrotum

When he went to a consult due to the impressive scrotal volume and penian deformity, the patient could not have sexual intercourse, could not maintain proper local hygiene and had walking problems. He required the administration of a treatment with antidepressants (**Fig. 1**).

Physical examination revealed a marked enlargement of the scrotum with the thickening and the loss of the elasticity of the peno-scrotal skin and subcutaneous tissue. Moreover, a little difference between the circumferences of the legs was visible, probably as a result of lymphatic disorder. The patient was in good condition without changes in laboratory tests.

**Fig. 2:**
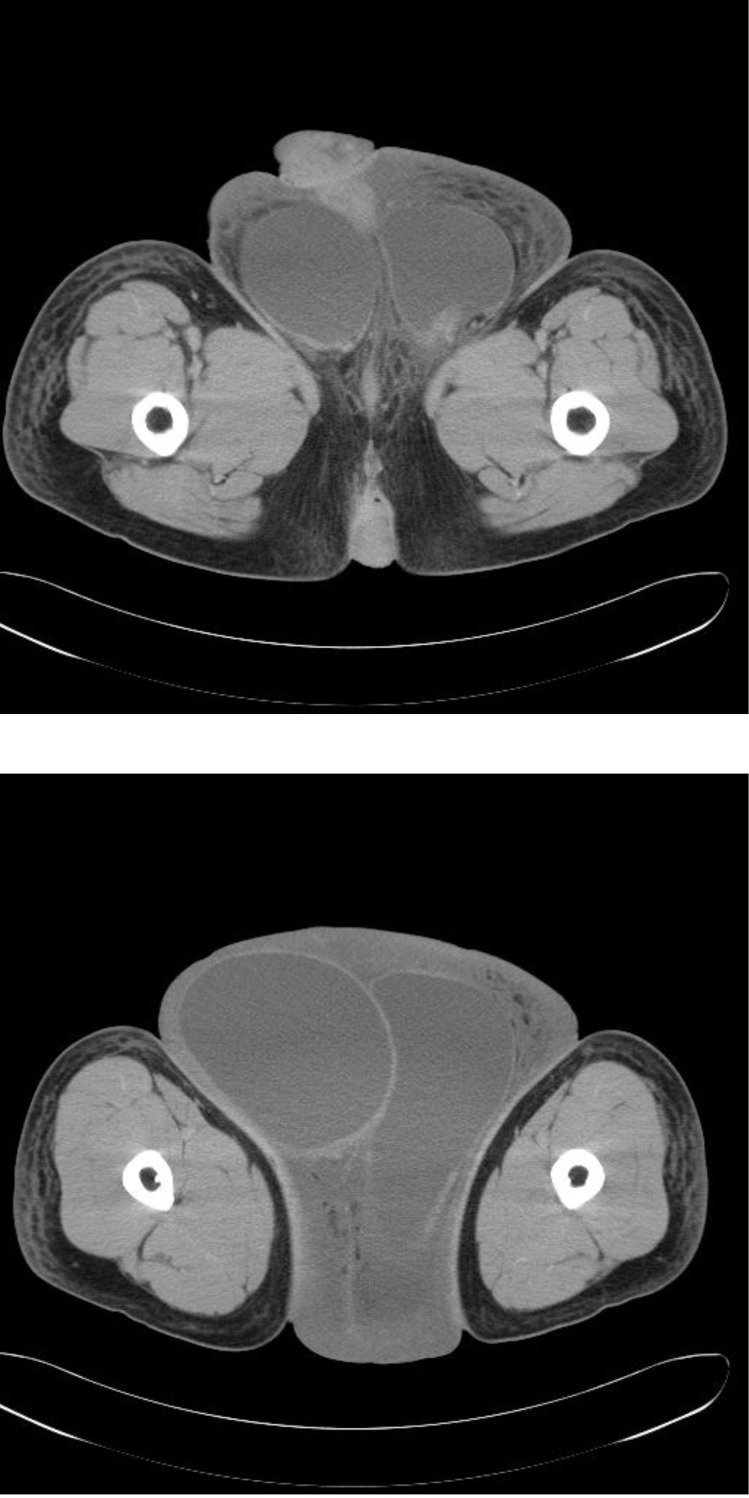
Computed tomography scan: scrotum enlargement of the scrotum

Abdominal and testicular ultrasound, chest and abdomino-pelvin CT did not show changes except for the global thickening of the peno-scrotal skin and subcutaneous tissue and important hydrocele on both sides (**Fig. 2**).

After a preoperative discussion with the patient (presenting him all the possible treatment options and related side effects) and considering important skin troficity changes, we decided to form a mixed surgical team urology – plastic and reconstructive surgery.

**The surgical technique**

Usual preoperative procedures were performed. After a spinal anesthesia the procedure began with the midline scrotal skin incision. Both vaginal testes and spermatic cords were isolated. Incision, excision and inversion of both vaginalis were performed; each vaginalis contained about 1,500mL of milky-like lymphatic fluid. The exceeding skin and subcutaneous tissue (with an infiltrated, boarded aspect) were removed. The penile skin was also excised immediately above Buck’s fascia. The remaining skin was used for the “new” scotum and a midline suture simulating the scrotal raphe was performed. The skinless penile shaft was covered with a split-thickness skin graft harvested from the anterior surface of the thigh. The scrotum was drained on both sides with a continuous drainage tube. An occlusive dressing consisting of a mild compression bandage which ensured adequate contact of the graft with the recipient bed and an indwelling urinary catheter were placed. 

**Fig. 3:**
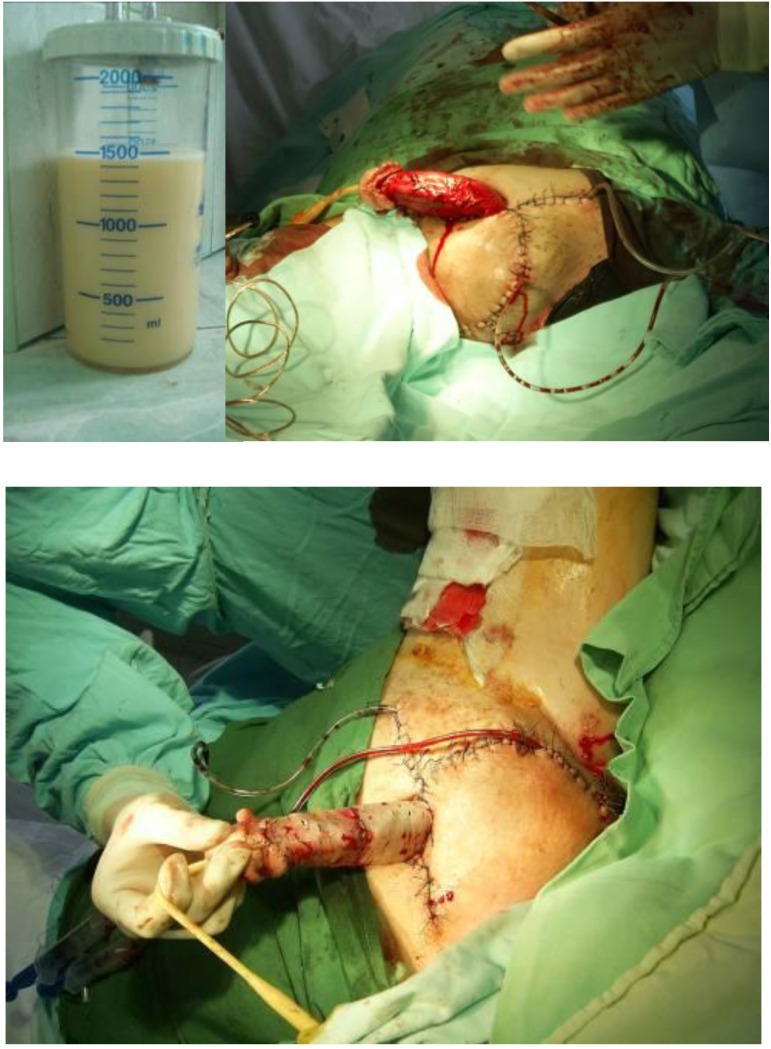
Intraoperative aspects

## Results

**Fig. 4:**
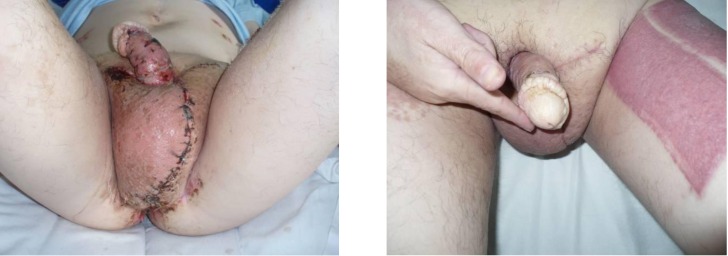
14 and 30 days postoperative aspects

Procedure and postoperative evolution were uneventful with minimal blood loss. The patient was dismissed after 14 days. The patient was evaluated after 7, 14, 30, 90 and 120 days (**Fig. 4**) without using a standardized questionnaire. A clear improvement of the aspect of the external genitalia and subsequent improvement of ambulation were observed. The improvement of sexual performance could not be directly assessed, although after a period of three months the patient declared that sexual intercourse became more effective with regard to penetration and satisfaction both for him and his partner. This was the result or the reason for a much better mental status of the patient.

## Discussion

Peno-scrotal lymphedema is, regardless of etiology, a clinical invaliding condition. Clinical treatment with different approaches has proven inefficient and is no longer used.

Surgical treatment based on the restoration of lymphatic drainage with different anastomoses is not used anymore due to poor results [**6**-**8**].

The excision of all the affected penile skin and subcutaneous tissue is a fundamental principle for an uneventful postoperative evolution and good plastic results [**5**].

Infections, dehiscence, or necrosis are few of the most unpleasant postoperative events that could happen. This is why the experience of the surgical team is very important. From this point of view the choice of a mixed surgical team turned into a win-to-win result as far as the patient and both surgical teams are concerned.

The coverage of the penile shaft with split-thickness skin graft is the optimal choice due to its very impressive cosmetic results. In the same time, the use of flaps could affect tactile sensitivity and erection, so, this technique has lost its supporters over time [**6**],[**8**].

The plastic surgery result is impressive when it is seen through the eyes of a patient with better mental status and recovered self-esteem.
